# Elevated Heart Rate Triggers Action Potential Alternans and Sudden Death. Translational Study of a Homozygous KCNH2 Mutation

**DOI:** 10.1371/journal.pone.0103150

**Published:** 2014-08-20

**Authors:** Ulrich Schweigmann, Peter Biliczki, Rafael J. Ramirez, Christoph Marschall, Ina Takac, Ralf P. Brandes, Dieter Kotzot, Zenawit Girmatsion, Stefan H. Hohnloser, Joachim R. Ehrlich

**Affiliations:** 1 Pediatric Cardiology, Innsbruck Medical University, Innsbruck, Austria; 2 Division of Clinical Electrophysiology, Goethe University, Frankfurt, Germany; 3 Center for Arrhythmia Research, University of Michigan, Ann Arbor, Michigan, United States of America; 4 Laboratory for Human Genetics, Martinsried, Germany; 5 Institute of Cardiovascular Physiology, Goethe University, Frankfurt, Germany; 6 Section for Human Genetics, Innsbruck Medical University, Innsbruck, Austria; 7 Div. of Cardiology, Deutsche Klinik für Diagnostik, Wiesbaden, Germany; University of Frankfurt - University Hospital Frankfurt, Germany

## Abstract

**Background:**

Long QT syndrome (LQTS) leads to arrhythmic events and increased risk for sudden cardiac death (SCD). Homozygous KCNH2 mutations underlying LQTS-2 have previously been termed “human HERG knockout” and typically express severe phenotypes. We studied genotype-phenotype correlations of an LQTS type 2 mutation identified in the homozygous index patient from a consanguineous Turkish family after his brother died suddenly during febrile illness.

**Methods and Results:**

Clinical work-up, DNA sequencing, mutagenesis, cell culture, patch-clamp, in silico mathematical modelling, protein biochemistry, confocal microscopy were performed. Genetic analysis revealed a homozygous C-terminal KCNH2 mutation (p.R835Q) in the index patient (QTc ∼506 ms with notched T waves). Parents were I° cousins – both heterozygous for the mutation and clinically unremarkable (QTc ∼447 ms, father and ∼396 ms, mother). Heterologous expression of KCNH2-R835Q showed mildly reduced current amplitudes. Biophysical properties of ionic currents were also only nominally changed with slight acceleration of deactivation and more negative V_50_ in R835Q-currents. Protein biochemistry and confocal microscopy revealed similar expression patterns and trafficking of WT and R835Q, even at elevated temperature. In silico analysis demonstrated mildly prolonged ventricular action potential duration (APD) compared to WT at a cycle length of 1000 ms. At a cycle length of 350 ms M-cell APD remained stable in WT, but displayed APD alternans in R835Q.

**Conclusion:**

Kv11.1 channels affected by the C-terminal R835Q mutation display mildly modified biophysical properties, but leads to M-cell APD alternans with elevated heart rate and could precipitate SCD under specific clinical circumstances associated with high heart rates.

## Introduction

Long QT syndrome (LQTS) is an inherited arrhythmogenic disease often caused by alterations in ion channels or cardiac structural proteins that prolong cardiac repolarization and render the heart susceptible to potentially lethal ventricular tachyarrhythmia. LQTS type 2 mutations are located in the pore-forming ion channel α-subunit Kv11.1 that underlies the rapid delayed-rectifier current (I_Kr_) and may cause loss of function through various cellular mechanisms, including modifications of biophysical properties or deficient trafficking of ion channels to the cell membrane [1], [2].

Individual delayed-rectifier gene mutations have different effects on patient outcome, but localization of LQTS mutations within the channel does not explain phenotypic variability among affected individuals, even within the same families. [2], [3] The clinical presentation may be individually modulated by the presence of specific circumstances and additional genetic modifiers such as mutations or single nucleotide polymorphisms (SNP) in other LQTS genes that affect repolarisation. [4], [5] Delayed-rectifier channels cluster with various anchoring proteins and chaperones that modulate trafficking and channel function. These may accordingly account for individual variability [6]–[8].

Mutations most commonly occur in the heterozygous state in patients with LQTS. Homozygous KCNH2 mutations have previously been reported as the “human HERG knockout” and typically translate into severe clinical phenotypes with greatly reduced ionic currents upon heterologous expression. [9] Some homozygous missense mutations are associated with cardiac structural abnormalities eventually leading to embryonic lethality [10].

Commonly, rest and arousal have been linked with arrhythmia occurrence rather than physical activity and elevated heart rate in patients with LQTS-2. [3] Febrile disease has been associated with ventricular arrhythmia in LQTS-2. [11] Mechanistically, Kv11.1 subunits are very sensitive to changes in temperature with increased current density upon elevated temperature, [12] but at the same time reduced membrane trafficking. [1] Furthermore, fever may increase dispersion of refractoriness and precipitate early after-depolarisations that can trigger polymorphic ventricular tachycardia (VT) and sudden cardiac death (SCD) [13].

The present study involved a detailed translational analysis of the previously unpublished KCNH2 mutation (p.R835Q) identified in the homozygous state in an index patient from a consanguineous Turkish family with LQTS-2 and a family history of SCD.

## Materials and Methods

### Patients, ECG measurements

A Turkish LQTS index patient and his family harbouring a previously unpublished C-terminal KCNH2 mutation was identified following an incident of SCD in the family. All family-members signed informed consent for genetic analysis regarding presence of the KCNH2 mutation. Cloning of the mutation and heterologous expression testing was approved by the ethics committee of Goethe-University, Frankfurt (254/05). Patient information was anonymized and de-identified prior to analysis. A large portion of the family lived in rural areas of Turkey and was not accessible for the purpose of expanding the family’s pedigree. QT intervals were manually measured from standard surface ECGs with help of a digital clickboard, and frequency correction was performed with Bazett’s formula.

### DNA sequencing and site-directed mutagenesis

Genomic DNA was extracted from EDTA blood, and coding exons of KCNQ1, KCNH2, SCN5A, KCNE1, KCNE2 genes were amplified using intronic primers. We have also screened SNPs specifically in NOS1AP.

### Cell culture and protein biochemical studies

Chinese hamster ovary (CHO) or human embryonic kidney (HEK) cells (Promochem, Wesel, Germany) were cultured with standard technique (at 37°C or 40°C) and transiently transfected with liposomal agents.

### Electrophysiological recordings

Currents were recorded from transfected CHO cells with whole-cell patch-clamp at 36±0.5°C (TC-324B temperature controller, Warner Instruments, Hamden, CT, USA) with an Axopatch 200B amplifier and pClamp 9.1 software (Molecular Devices, Ismaning, Germany).

### In silico analysis

Computational analysis of the effects of the KCNH2-R835Q mutation on cardiomyocyte electrophysiology was performed in silico using a mathematical model of the human ventricular action potential (AP) [14], [15].

### Statistical analysis

Data were analyzed with Clampfit (Molecular Devices, Ismaning, Germany) and GraphPad Prism (GraphPad Software, San Diego, CA, USA). Patch-clamp and protein biochemical data are presented as mean±SEM. A two-tailed p<0.05 obtained with Student’s t test was considered to indicate statistical significance.

(For more detailed description please view the additional Methods S1).

## Results

### Patient population, DNA analysis and heterologous mutation expression

**Figure 1:**
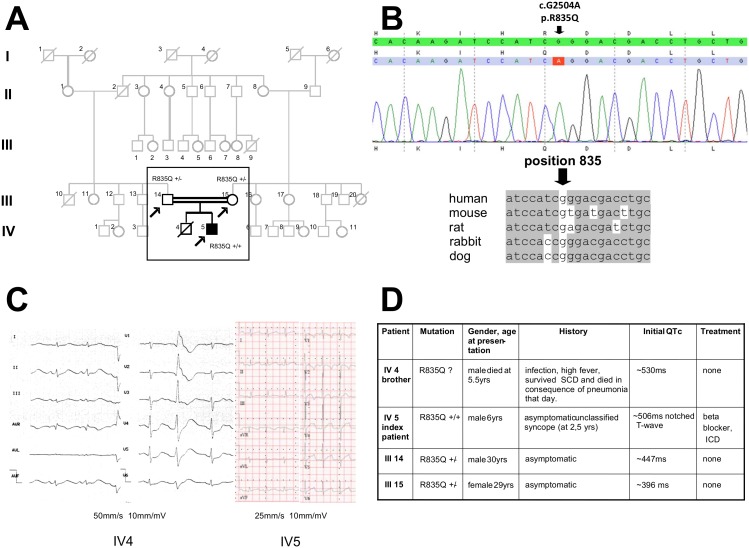
Pedigree and ECGs of the index patient and his deceased brother. **A,** arrows indicate genotype-positive family members; filled black square represents the index patient. Grey symbols represent ungenotyped family members. **B,** DNA sequence chromatograms indicating homozygous KCNH2 mutation in the index patient. The mutation is located on the C-terminus of the KCNH2 gene, causing a guanine to alanine change (c.G2504A, p.R835Q) at a conserved location. **C,** ECG of the index patient and his brother at initial presentation. Paper speed and leads of ECGs are indicated. The index patient (IV 5) had a QTc of ∼506 ms with notched T waves. **D,** illustrates patient characteristics. Yrs.: years; SCD: sudden cardiac death.

We performed a detailed translational analysis of a consanguineous Turkish family with LQTS-2 in order to study genotype-phenotype correlations of the previously unpublished KCNH2 mutation c.2504G>A; p.835R>Q was identified in the index patient whose brother died suddenly. Parents were I° cousins and heterozygous for the mutation (Fig. 1A). QTc intervals were ∼447 ms in the father and ∼396 ms in the mother. The index patient’s DNA chromatograms of KCNH2 sequencing are presented in Fig. 1B, illustrating the location of the mutation within the KCNH2 DNA sequence and within a schematic protein structure with amino acid localization. The brother was asymptomatic until the age of five years, when he developed high-grade fever and was resuscitated following ventricular fibrillation cardiac arrest. His admission ECG showed prominent QT prolongation and the patient died in consequence of pneumonia and hypoxia on the day of admission. His then 2-year-old brother was asymptomatic and showed a prolonged QT interval (∼506 ms) with notched T waves upon family screening (Fig. 1C). He had unclassified syncope at 2.5 years of age and suspected cardiac syncope at 5.9 years of age despite beta-blocker treatment. Following the second event, he received an implantable cardioverter defibrillator (ICD). Characteristics and history of family members are summarized in Fig. 1D.

The mutation was classified as “possibly damaging” by means of PolyPhen2 (score 0.897); SIFT detected the mutation as “deleterious” with a probability of 0.03 (deleterious: probabilities <0.05), and Mutation Taster classified the mutation as “disease-causing”, p: 0.999.

### Electrophysiological characterization

**Figure 2:**
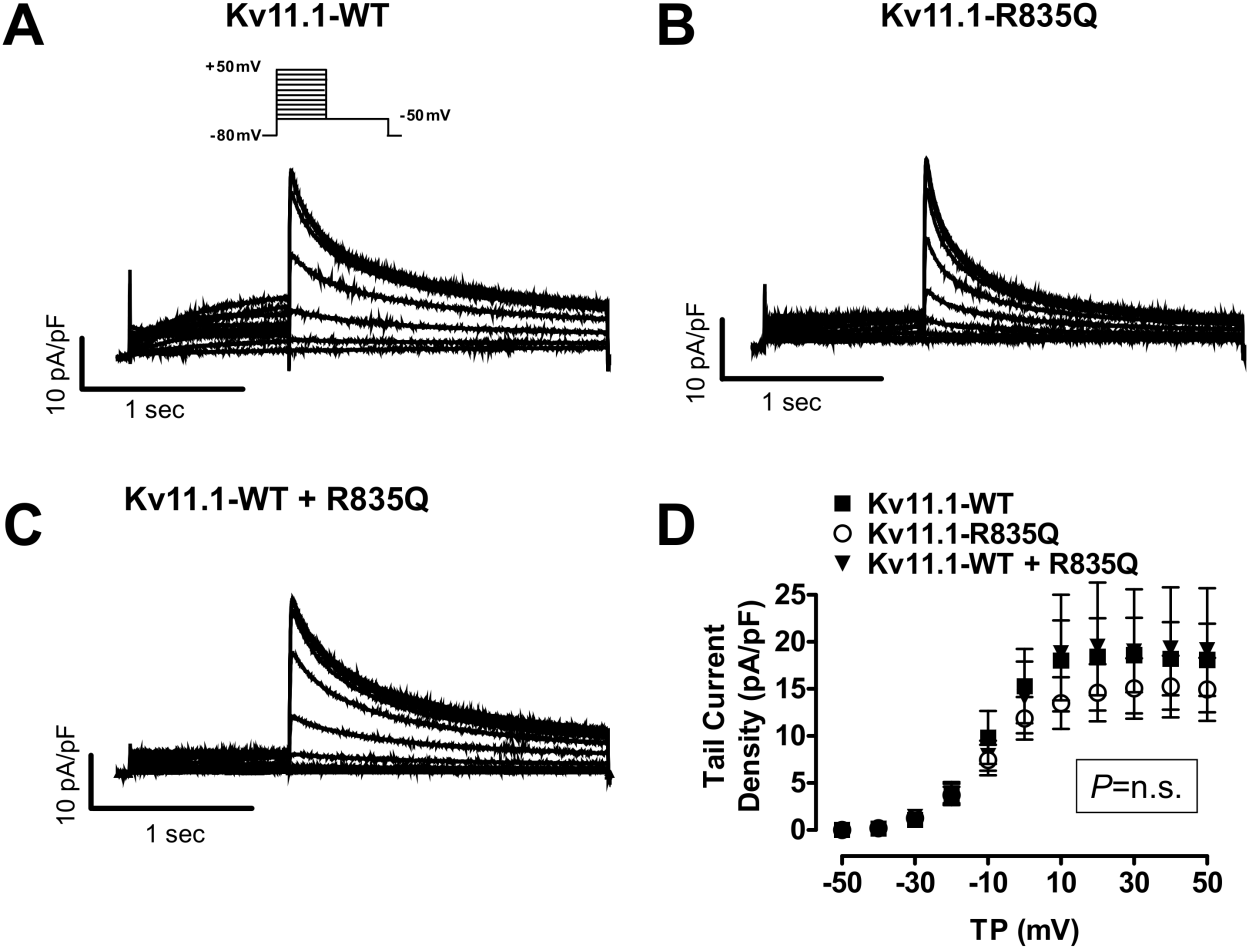
Representative I_KCNH2_ recordings (protocol in inset A) obtained from CHO cells transiently transfected with KCNH2-WT and KCNH2-R835Q cDNA (A–B). Currents recorded from cells with Kv11.1-R835Q (n = 13 cells) or Kv11.1-WT with R835Q (n = 9) were similar in size to the tail currents from cells transfected with Kv11.1-WT (n = 13, C). D, mean±SEM deactivating tail current density recorded upon repolarization to −50 mV. Ordinate and abscissa scale bars represent 10 pA/pF and 1 sec, respectively, for all current examples. WT: wild type; n.s.: not significant; TP: test potential.

Currents evoked from CHO cells transiently transfected with KCNH2-wild type [WT] and KCNH2-R835Q cDNA showed only nominally decreased I_kv11.1_ density with mutant channels (at +50 mV: WT 18±4 pA/pF, R835Q 15±3 pA/pF, p = n.s. n = 13 cells each, Fig. 2A–B). At test potentials above −10 mV, mutant I_Kv11.1-R835Q_ showed 20.8% less current than did WT (p = n.s.). In order to mimic heterozygosity of the clinical cases we compared cells transfected with ∼1.0 µg WT KCNH2 cDNA and cells co-transfected with ∼0.5 µg WT and ∼0.5 µg of R835Q mutant cDNAs. Analysis of charge carried by currents of various expression groups over time was consistently reduced in a nominal manner. Tail currents from cells transiently transfected with KCNH2-R835Q carried approximately 25% less charge at repolarizing test pulses from +50 mV than cells transfected with KCNH2-WT (208±50 pC vs. 259±48 pC, p = n.s.).

Currents recorded from cells co-transfected with KCNH2-WT and KCNH2-R835Q showed tail currents similar in size to those of cells co-transfected with KCNH2-WT (upon repolarization from +50 mV) to 19±6 pA/pF (n = 9), versus 18±4 pA/pF for KCNH2 alone (n = 13, p = n.s, Fig. 2C). Step currents were also similar. For instance, depolarization to 0 mV generated I_kv11.1_ of 4.1±0.8 pA/pF for WT (n = 13) in comparison to 2.3±0.5 (n = 13), 2.9±0.7 pA/pF (n = 9, p = n.s) for KCNH2-WT, KCNH2-R835Q mutations and WT+R835Q co-transfection, respectively. Biophysical analysis of ionic current properties also showed slight acceleration of deactivation and more negative V_50_ (−14.9 mV [WT] vs. ∼−9.4 mV [R835Q], n = 13 each, p = n.s., Fig. 2A).

**Figure 3:**
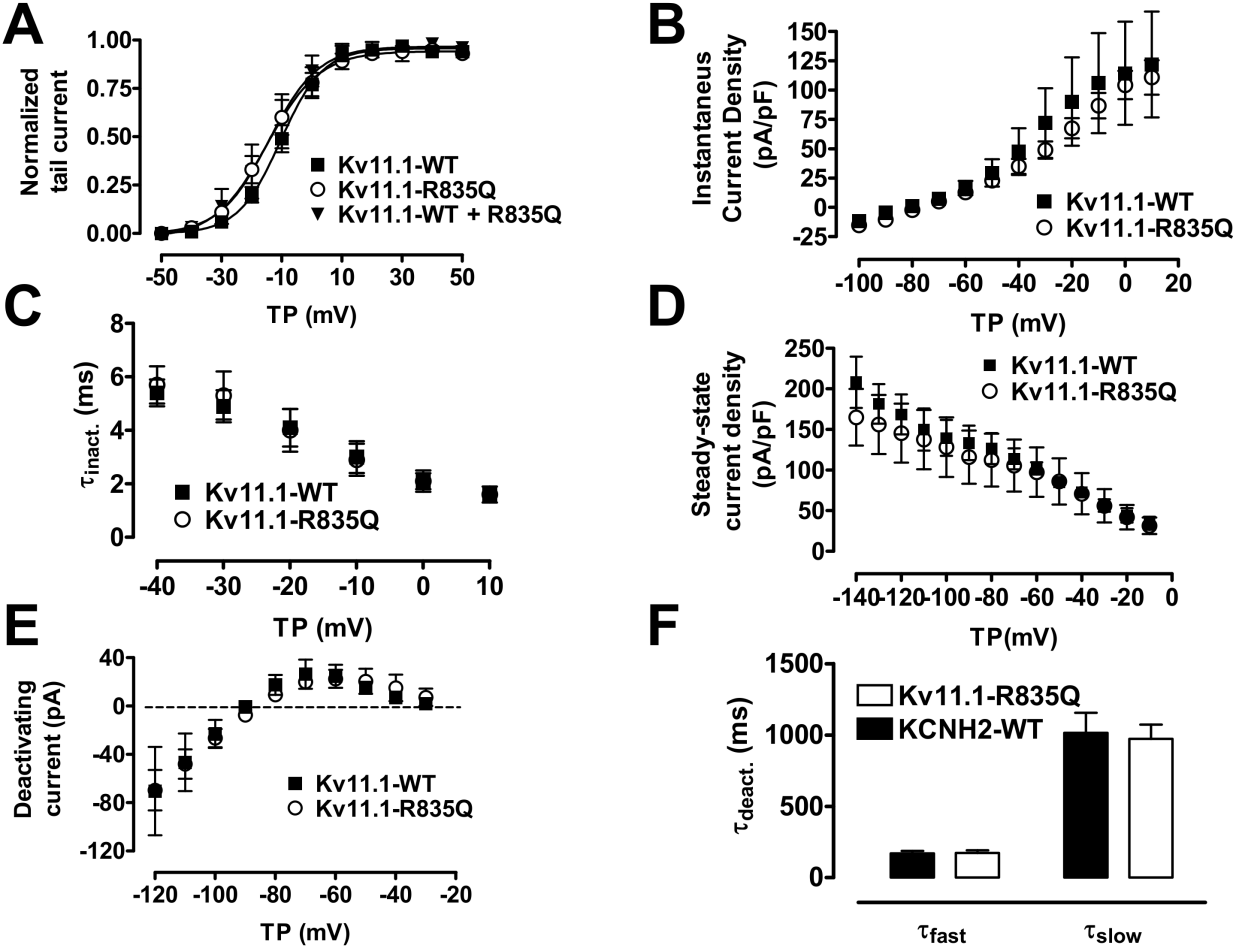
**A**, normalized tail currents illustrating voltage dependence of Kv11.1 current activation. Fits correspond to Boltzman functions (A = A_0_/(1+exp[(V_50_−V)/S], with A = current at potential V, A_0_ = maximal normalized tail current, S = slope factor) to mean±SEM data. Half-activation voltage (V_50_) was slightly more negative (∼−9.4 mV vs. ∼−14.9 mV, n = 13 each, P = n.s.) for Kv11.1-R835Q. Instantaneous inactivation-free current did not differ (**B**), and inactivation kinetics obtained with monoexponential fits (y = A*exp(−t/τ)+C; y = deactivating tail current at time point t, A = amplitude constants, τ = time constants, C = steady-state current) were also similar between groups (**C**). **D**, no difference was detected between steady-state inactivation-free currents. **E**, mean±SEM current-voltage relationship for deactivating currents. Fillustrates mean±SEM time constants (τ) obtained from bi-exponential fits (y = A_1_*exp(−t/τ_1_)+A_2_*exp(−t/τ_2_)+C; y = deactivating tail current at time point t, A_1_, A_2_ = amplitude constants, τ_1_, τ_2_ = time constants, C = steady-state current) to deactivating tail currents, R835Q did not exhibit overall accelerated deactivation kinetics. White bars correspond to mutant and black bars to wild-type. Abbreviations as above.

Deactivation kinetics were studied by fitting bi-exponential functions to currents returning to −50 mV from +50 mV. I_kv11.1_ deactivation time constants were not significantly altered by co-expression of mutant cDNA. Respective time-constants were: Kv11.1-WT (n = 13) 1015±140 (τ_1_), 171±17 ms (τ_2_), Kv11.1-R835Q (n = 13) 973±100 (τ_1_), 173±19 ms (τ_2_;) and Kv11.1-WT plus Kv11.1-R835Q: 1115±76 (τ_1_), 179±29 ms (τ_2_, n = 9, p = n.s., Fig. 3E).

Assessment of inactivation-free current showed linear I–V relationships for mutant construct and was not significantly smaller for Kv11.1-WT or Kv11.1-R835Q (at 0 mV: 114±44 pA/pF for WT; 104±12 for R835Q, p = n.s. vs. WT Fig. 3D). Mono-exponential time constants of inactivation were also not significantly altered (5.4±0.5 ms for Kv11.1-WT vs. 5.7±0.7 for Kv11.1-R835Q, p = n.s. for all vs. WT, Fig. 3F).

### Molecular biology studies and confocal microscopy

**Figure 4:**
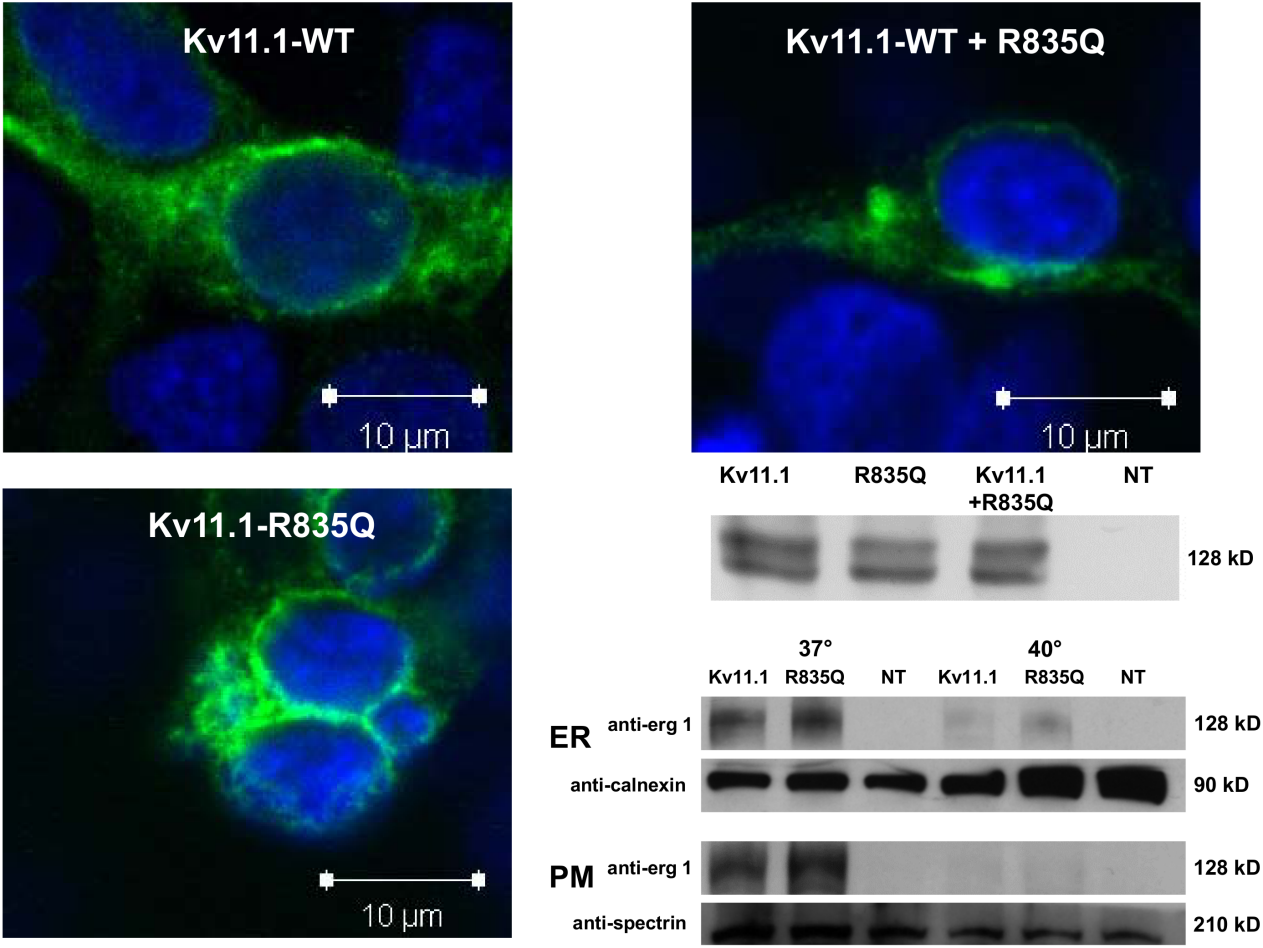
**Panels A–C,** representative images obtained from confocal microscopy of transiently transfected HEK cells. R835Q mutant channels do not appear differently distributed in comparison to WT KCNH2. **D**, Immunoblots using anti-erg1 (2, 5 µg/mL) of crude membrane extracts from heterologous expression in HEK cells, indicating equal protein expression level. Illustrated below are endoplasmic reticulum and plasma membrane fraction with respective markers of equal protein loading (calnexin for endoplasmic reticulum, spectrin for plasma membranes). Exemplary Western blots of preparations at physiological temperature (37°C) and 40°C (to simulate febrile illness of the index patient’s brother) are shown. No differences were observed in Kv11.1-WT or Kv11.1-R835Q plasma membrane representation of the two proteins under the two conditions. ER: endoplasmic reticulum fraction; PM: plasma-membrane fraction; WT: wild type; NT: non-transfected cells.

In order to perform detailed analyses of Kv11.1-R835Q channel trafficking properties, we studied cellular trafficking of α-subunit proteins by confocal microscopy. Consistently with the electrophysiological experiments, no difference in Kv11.1 membrane expression was seen between WT and R835Q channels (Fig. 4).

Results from immunoblots obtained after heterologous expression of WT and mutant ion channels yielded protein bands at 135 and 155 kD for immature and fully glycosylated ion channels with similar overall expression and distribution patterns of WT and mutant channels (Fig. 4). Studies of membrane proteins at 37°C and 40°C (mimicking the febrile status of the index patient’s deceased brother) did not provide evidence for altered membrane expression of the Kv11.1 mutation under any condition.

### In silico analysis

Expression of the KCNH2-R835Q mutation revealed that the biophysical properties were nominally different from those of wild type. To quantitatively assess the impact of these subtle differences on human AP morphology and duration, computational analysis was performed in silico using a mathematical model of the human ventricular AP. [14], [15] The ratio of R835Q:WT whole-cell Kv11.1 current at test potentials between −10 and +50 mV averaged 79.2%. Thus, simulations of R835Q-mutant APs incorporated a reduction in I_Kr_ whole-cell conductance by 20.8% of WT values. Similarly, the ratio of R835Q:WT activation time constant was 0.875. Thus, simulated mutant APs used an activation time constant that was 12.5% lower than that of WT. Electrophysiological characterization showed a leftward shift of activation by 5.53 mV and an increase in slope factor of 11.9%; both of these were implemented to simulate R835Q-mutant APs.

**Figure 5:**
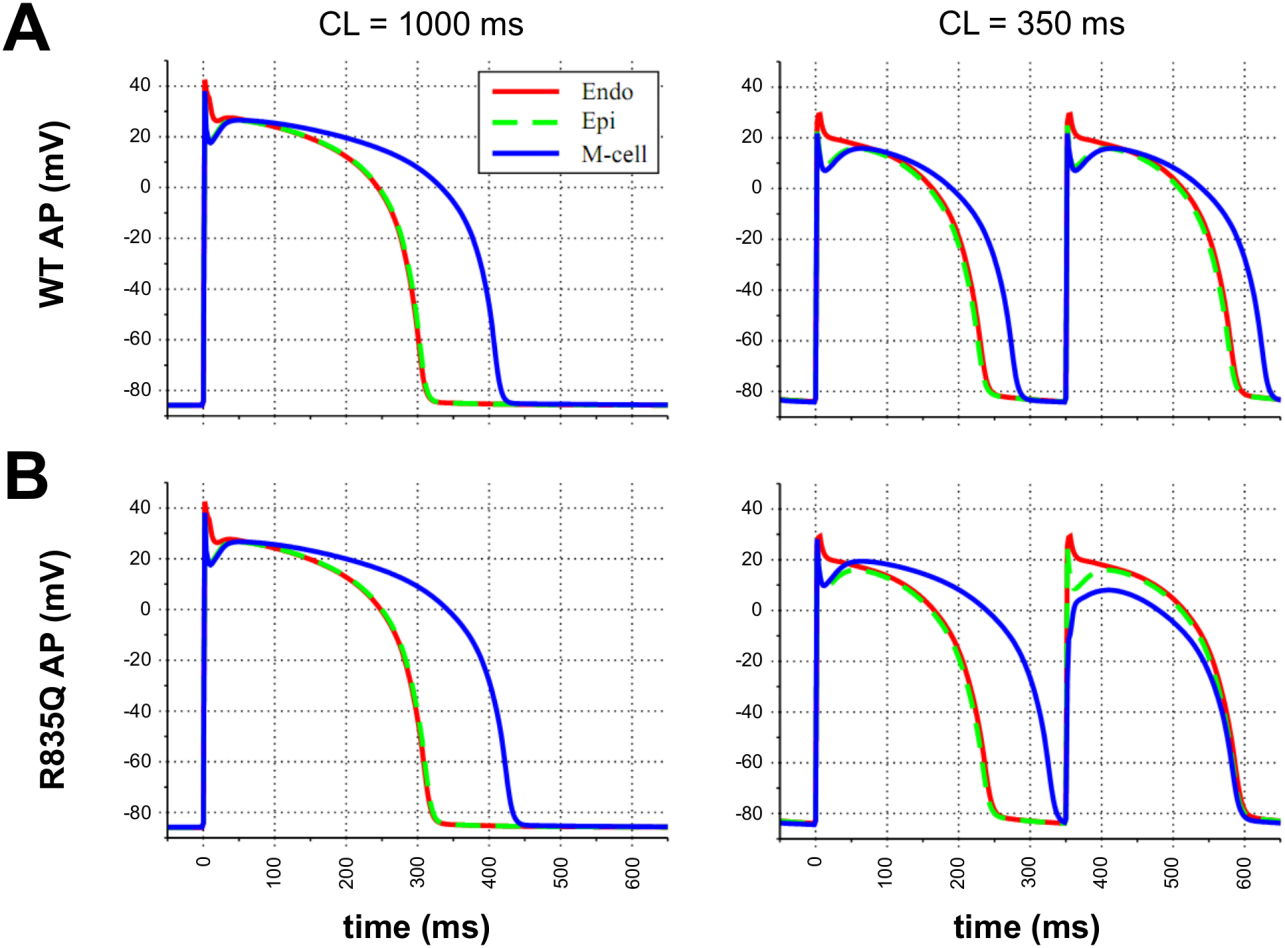
In silico analysis of R835Q mutation in human ventricular AP model (ten Tusscher model). Simulated human ventricular model of (**A**) WT and (**B**) R835Q action potentials (APs) at cycle lengths (CL) of 1000 and 350 ms. The R835Q mutation has a greater impact on M-cell AP morphology than on that of epicardial or endocardial cells. At CL 1000 ms, M-cell APD_90_ is prolonged from 412 ms in WT to 428 ms in R835Q mutants, while epi/endo APD_90_ values are nominally prolonged from 308/306 ms to 316/314 ms, respectively. At CL 350 ms, epi/endo APD_90_ values increase from 232/236 ms in WT to 240/242 ms in mutant; M-cell APD_90_ is stable at 278 ms in WT, but displays APD alternans in R835Q-mutant APs with APD_90_ values of 330/236 ms.

In this human ventricular AP model, epicardial and endocardial AP waveforms are very similar with APD_90_ values of 308 and 306 ms at a stimulation cycle length (CL) of 1000 ms, and 232 and 236 ms at CL 350 ms. Simulated M-cell APs have longer APD_90_ values of 412 and 278 ms at CL 1000 and 350 ms (Fig. 5A). Incorporation of R835Q properties into the calculation of I_Kr_ in the AP model showed that mutant APD_90_s were slightly prolonged at CL 1000 ms as compared to WT in epicardial (316 ms, 2.6% prolongation vs WT), endocardial (314 ms, 2.6% prolongation), and M-cells (428 ms, 3.9% prolongation). Increasing the stimulation pacing rate to CL 350 ms produced APD alternans in M-cells, but not in epicardial or endocardial cells (Fig. 5B). At CL 350 ms, M-cell APD_90_ alternated between long and short values of 330 and 236 ms. APD alternans remained quantitatively stable in M-cells, cycling between these long and short values for the duration of stimulation at CL 350 ms (over 60 beats).

**Figure 6:**
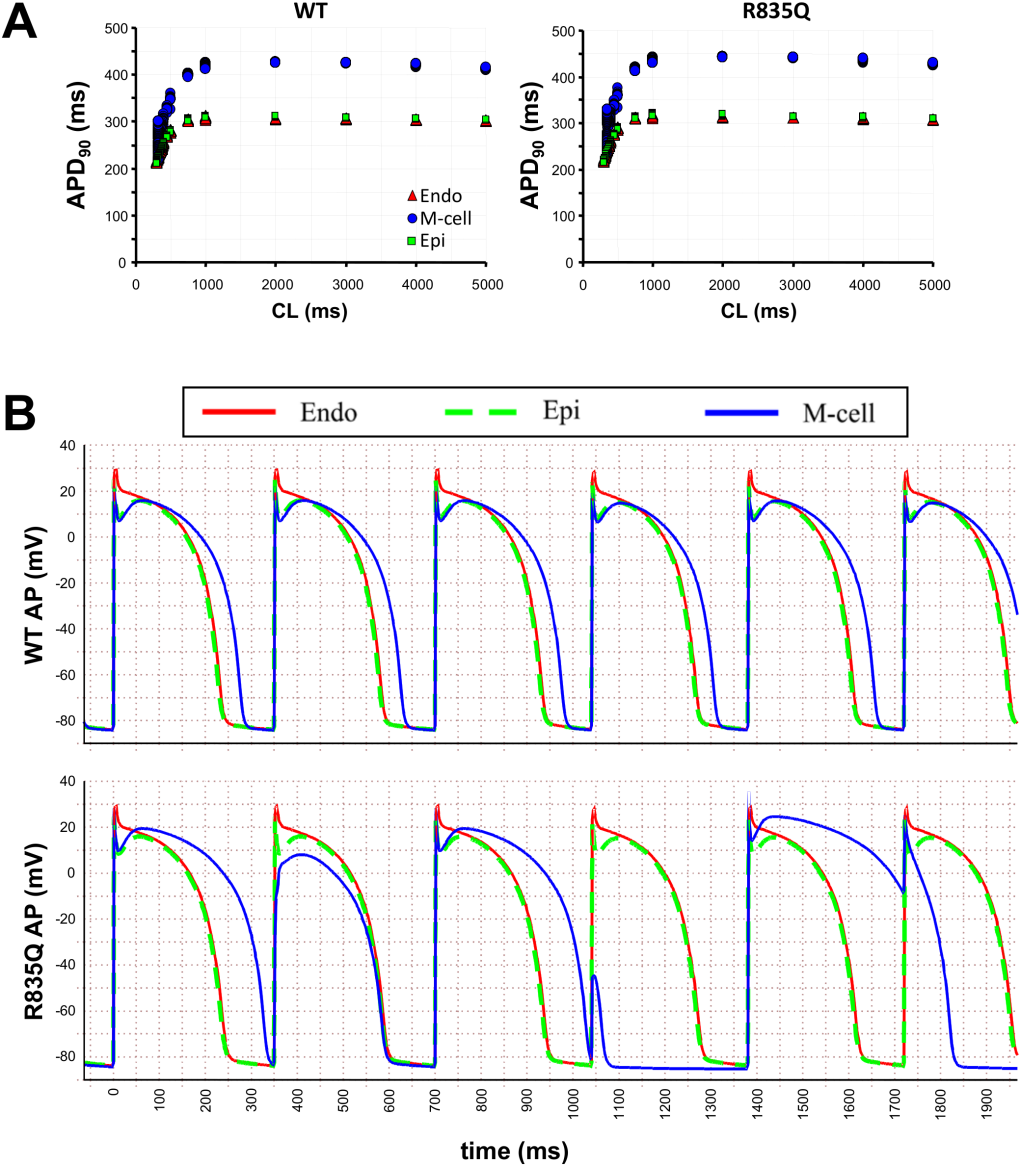
Dynamic restitution and failure to capture at fast rates in R835Q-mutant AP model. (**A**) Dynamic restitution curves for WT and R835Q show that APD_90_ is moderately increased in R835Q as compared to WT. APD prolongation and restitution steepening are most pronounced in ventricular M-cells. (**B**) Stimulation at 350 ms induces APD alternans in mutant M-cell but not WT APs. As stimulation CL decreases from 350 to 340 ms, APD_90_ is stable in WT M-cells; however, in R835Q-mutant M-cells subsequent stimulation encroaches on the refractory period of the previous long AP, failing to capture.

A dynamic pacing protocol was implemented to assess dynamic restitution curves in WT and R835Q models. Despite the slightly faster and earlier activation kinetics of I_Kr_ in R835Q-mutant APs, the reduction in whole-cell I_Kr_ conductance prolonged APD across all CLs examined, shifting dynamic restitution curves slightly upward (Fig. 6A). Prolongation of APD was greater for M-cells than for epicardial or endocardial cell types, and maximal slope was higher in R835Q than in WT. While a pacing CL of 350 ms produced stable alternans in M-cells, further decreasing CL to 340 ms failed to elicit an AP (depolarization upstroke <−40 mV) when the CL encroached on the preceding APD (Fig. 6B). Thus, biophysical properties of the R835Q mutation produce nominal APD_90_ prolongation at a stimulation CL of 1000 ms and slower; prolongation of mutant APs is more pronounced in M-cells.

## Discussion

### Main findings

The results of our study provide evidence that Kv11.1 channels affected by the C-terminal R835Q mutation display mildly modified biophysical properties, but may cause an arrhythmic substrate with AP prolongation at rest and M-cell APD alternans at elevated heart rate. Changes in ionic current properties were mild but nevertheless caused relevant QT prolongation. Most probably, it was for this reason that the index patient did not have a deleterious phenotype, as otherwise described for homozygous KCNH2 mutations.

The sum of biophysical alterations led to significant proarrhythmic substrate. Despite the slightly faster and earlier activation kinetics of I_Kr_ in R835Q-mutant currents, that would be consistent with more I_Kr_ available for repolarisation, the reduction in whole-cell I_Kr_ conductance prolonged APD in the computer model and shifted dynamic restitution curves upward. Fever has previously been associated with malignant ventricular arrhythmia in patients and experimental models of LQTS type 2. No difference in Kv11.1 protein expression was documented at elevated temperature. This study proposes elevated heart rate as an additional modulator of the arrhythmogenic substrate in LQTS type 2. These results are of potential interest as LQTS-2 is not typically associated with rapid heart rate-related arrhythmia due to intact I_Ks_ [3].

### Homozygous KCNH2 mutations

In patients with LQTS type 2, mutations are most commonly present in a heterozygous state. A first description of a homozygous KCNH2 mutation (illustrated as the “human HERG knockout”) was provided by Hoorntje et al. [16] The mutation consisted of a duplication of base-pairs 558 to 600 in exon 4 of the KCNH2 gene, resulting in frame shift and premature truncation of the protein, leaving a non-functional Kv11.1 fragment. The authors described a female index patient who developed Torsades-de-pointes tachycardia shortly after birth. She was treated with betablockers, magnesium and pacing and remained well during follow-up. Interestingly, the patient’s mother experienced a stillbirth at week 36 with intrauterine fetal heart failure and hydrops. A pathological cardiac phenotype was apparent in endo- and myocardial tissue of the fetus. Another study in mice introduced a homozygous mutation (N629D) into the orthologous mERG gene and illustrated that loss of I_Kr_ function was associated with disturbed development of the right ventricle, bulbus cordis, and pharyngeal arches. [10] Such dysmorphic features have not been described in other (human) cases including our report.

Lupoglazoff et al. have previously reported the mutation Kv11.1 R835W in neonates with 2∶1 atrioventricular block and severe ventricular arrhythmias. Three mutations were identified: one in KCNQ1 and one in SCN5A, both inherited from the mother, and the third in KCNH2 inherited from the father [17].

Other homozygous patients with a KCNH2 mutation (p.L552S, located at the C-terminus) were described by Piippo et al. in a Finnish population. [18] These authors found two siblings with homozygous KCNH2 mutation presenting with a severe clinical phenotype including aborted cardiac arrest and AV nodal block. Interestingly, in this study parents were heterozygous and asymptomatic carriers of the mutation. In both parental families QT intervals differed significantly between symptomatic and asymptomatic heterozygous mutation carriers (500±59 vs. 412±34 ms, respectively). This suggests, in parallel to the family described by us, that additional factors may importantly modulate repolarization. A similarly severe phenotypic appearance was described for another homozygous KCNH2 missense mutation, namely p.R752Q, which in the homozygous state resulted in almost complete loss of functional I_Kr_, causing a severe arrhythmogenic phenotype. [9] In that population, heterozygous mutation carriers were similarly clinically unremarkable. There were no additional mutations or known SNPs such as K897T in KCNH2 or in NOS1AP [5], [19] in the family described here. This does not rule out the presence of modifiers in other as yet undefined locations in the index patient or his deceased brother.

### Genotype-phenotype correlation in LQTS-2

The results of our study suggest that KCNH2 channels affected by the C-terminal p.R835Q mutation display only mildly modified biophysical properties, which may nevertheless precipitate QT prolongation and SCD under specific circumstances. Given the mild cellular phenotype of the mutation described in our report and the fact that parental mutation carriers did not exhibit an LQTS phenotype, one might argue that this mutation is not disease-causing. Unfortunately, the family was not amenable to further clinical or genetic studies of its members as a large portion of the family lived in rural Turkey. Accordingly, segregation of the mutation cannot be studied by clinical presentation, and the possibility to perform DNA analysis on the deceased child was limited because of tissue degradation caused by formalin fixation. However, calculation of the functional effect of KCNH2-R835Q with PolyPhen 2 predicted this variant to be “possibly damaging”, PSIC score difference: 1.754. This might be related to a strong conservation of the amino acid sequence in various animal species.

We previously reported the in vitro characteristics of three C-terminal KCNH2 missense mutations located in close proximity. The clinical presentation of respective index patients was very heterogeneous. We found that mutations affecting adjacent amino acids may lead to very different cellular characteristics, and the clinical presentations of index patients are similarly divergent. These findings provide evidence that at the present time even a detailed characterization of LQTS mutations does not directly help with clinical risk stratification. [2] Nevertheless, such work is important to fully understand ion channel behaviour.

### Mechanism of fever-induced changes

In addition to numerous previous case reports naming fever as the arrhythmia trigger in Brugada syndrome or LQTS type 2, Amin et al. reported on a family with fever-accentuated QT prolongation and polymorphic ventricular tachycardia during febrile disease. [11] These authors documented shortened QTc in healthy probands, but a linear increase in QTc with temperature rise in patients. This study convincingly illustrated reduced expression of KCNH2 subunits with elevated temperature, linking it with arrhythmia precipitation. Another study in canine ventricular wedges documented an additional pro-arrhythmic increase in transmural dispersion of refractoriness under experimental conditions of reduced I_Kr_ density. [13] This led to prominent triggering of early after-depolarizations. Our study adds a potential role of elevated heart rate together with nominally changed biophysical properties to the list of contributors to arrhythmogenic responses.

### Arrhythmogenic role of M-cells

The transmural dispersion of repolarization is caused by intrinsic differences in APD of different cardiomyocyte populations (epicardial, M-cells and endocardial cells) of the ventricular myocardium. M-cells are also present in the deep cell layers of endocardial structures, including papillary muscles, trabeculae and the interventricular septum. M-cells show greater APD prolongation and develop early after-depolarizations in response to I_Kr_-blockers, whereas epicardium and endocardium are generally less likely to do so. [20] M-cells also more rapidly develop delayed after-depolarizations in response to agents that increase calcium load in cardiomyocytes. The ionic basis for these features of M-cells includes the presence of a smaller slowly activating delayed-rectifier current (I_Ks_), larger late sodium current (late I_Na_), and a larger Na^+^/Ca^2+^ exchanger current. [21] Accordingly, M-cell repolarization depends more on I_Kr_. There are only few data dealing with the presence of M-cells in the human heart. Glukhov et al. provided evidence in agreement with animal studies showing that deceleration-induced prolongation of APD in nonfailing hearts was much greater in the mid-myocardial area than in epicardial or endocardial regions [22].

In our study using an established human AP computer model, mild reduction in repolarization reserve due to alteration of I_Kr_ parameters led to AP alternans of M-cells, which is a substrate for the development of ventricular arrhythmia. [23] Susceptibility to macroscopic T wave alternans is a hallmark of patients with LQTS [24], and reductions in I_Kr_ have been linked to its occurrence. Conversely, overexpression of I_Kr_ can effectively suppress alternans. [25] The increase in dynamic AP restitution introduced by the mutation will enhance susceptibility to alternans formation and subsequent arrhythmia.

## Conclusions

In this study we have characterized a novel C-terminal mutation in KCNH2 (p.R835Q) detected in the homozygous state in the index patient with QT interval prolongation. His brother died suddenly during febrile illness. Detailed cellular electrophysiological studies found only mild differences between WT channels and mutation. There was slightly more rapid activation of mutant I_Kr_, and half-activation voltage was nominally shifted to more negative potential, which should lead to increased I_Kr_ availability while overall current amplitude was reduced. There were no differences in trafficking or cellular distribution. In silico modeling found AP prolongation and alternans at CL of 350 ms with failure to elicit APs at still higher frequencies. While T wave alternans has been a hallmark of LQTS for decades, elevated heart rate is an unusual mechanism precipitating arrhythmia in patients with LQTS type 2 and unexpected in clinically severe phenotypes.

## Supporting Information

Methods S1 The supplemental methods section contains detailed description of experimental procedures.
(DOC)
