# Supplementary material for: Elevated Heart Rate Triggers Action Potential Alternans and Sudden Death. Translational Study of a Homozygous KCNH2 Mutation
Source: PLoS One. 2014 Aug 20;9(8):e103150. doi: 10.1371/journal.pone.0103150 (PMC4139196; doi:10.1371/journal.pone.0103150)
Supplement: Methods S1 — The supplemental methods section contains detailed description of experimental procedures. (DOC) [file pone.0103150.s001.doc]

**METHODS S1**

**DNA sequencing and site-directed mutagenesis**

Sequencing reactions were automatically analysed. KCNH2 constructs were created by overlap extension PCR technique (numbering according to NM_000238). Primers used for mutagenesis are provided in Table 1. All PCR-generated sequences were verified by double-stranded sequencing.

Analysis of PCR products for their disease potential was performed with dedicated programs. MutationTaster employs a Bayes classifier to predict the disease potential of an alteration.[1] PolyPhen-2 is a further development of the PolyPhen2 tool for annotating coding nonsynonymous SNPs. The functional importance of an allele replacement is predicted from its individual features by means of a naive Bayes classifier.[2] SIFT predicts whether an amino acid substitution affects protein function based on sequence homology and the physical properties of amino acids.[3]

**Cell culture and protein biochemical studies**

Proteins were obtained from HEK cells 48 hours post-transfection as follows: all steps were performed at 4°C and (mg/ml) benzamidine 10, leupeptine 5, trypsin inhibitor 5 were added as protease inhibitors. Cells were lysed in 10ml buffer containing (mmol/L) Tris-HCl (pH7.4) 5, EDTA 2 and PMSF 1. After polytron homogenization, lysates were centrifuged at 1000xG (5 min) and the resulting supernatant was centrifuged at 28.000xG (20 min). The pellet contained crude membranes. Proteins were transferred to nitrocellulose membranes and incubated with anti-Kv11.1 antibody (anti-erg1, ICS, Munich, Germany, 2,5µg/mL). Bands were visualized by chemiluminescence.

Green-fluorescent protein (GFP, 0.1µg) served as transfection marker for electrophysiological recordings.

**Electrophysiological recordings**

Borosilicate glass electrode tips had 1.5-2.5 MΩ resistances when filled with internal solution. Capacitances averaged 31±3 pF (n=35 cells). Junction potentials averaged 3.9±0.2 mV, and voltage compensation for junction potentials was not performed. The extracellular solution contained (mmol/L) NaCl 132, KCl 4.7, MgCl_2_ 1, CaCl_2_ 1, NaH_2_PO_4_ 0.36, HEPES 10 and dextrose 5 (pH 7.4 with NaOH). Internal solution for current recordings contained (mmol/L) KCl 110, K_2_-ATP 5, MgCl_2_ 1, HEPES 10, EGTA 5, (pH 7.3 with KOH). Currents were recorded with 1-sec depolarizations from a holding potential of –80 mV (0.08 Hz), with deactivation observed during 2-sec repolarizations to –50 mV.[4] Voltage dependence of Kv11.1 current (I_Kv11.1_) deactivation was assessed with 400-ms pulses to +40 mV followed by 2-sec repolarizations to voltages between -120 and 0 mV. The current-voltage relationship of inactivation-free I_KCNH2_ was evaluated by following a 500-ms activating step to +40 mV by a 25-ms pulse to -110 mV and then by 500-ms test pulses to potentials between -80 and +60 mV. Steady-state I_KCNH2_ inactivation was assessed with 500-ms pulses to +40 mV for 500 ms followed by 25-ms steps to between -140 mV and 0 mV and then by 500-ms depolarizations to +40 mV.[4,5]

**Confocal microscopy**

Transiently transfected cells were grown on sterile plastic coverslips (ibidi, Martinsried, Germany) for 48 hours. Cells were fixed with 2% paraformaldehyde (Merck, Darmstadt, Germany), washed with PBS and blocked with 5% donkey serum (Dianova/Jackson, Hamburg, Germany), 5% bovine serum albumin (BSA, Carl Roth, Karlsruhe, Germany) and permeabilized with 0.2% Triton X-100 (Sigma-Aldrich Biochemie GmbH, Hamburg, Germany). Cells were incubated overnight (4°C) with primary antibodies (1/200 for all antibodies used) and washed with phosphate-buffered saline. Secondary antibody anti-rabbit IgG Alexa-fluor-555 (Molecular Probes Inc., Eugene, OR, USA) was incubated for one hour. We also used 4',6-diamidino-2-phenylindole (DAPI) for DNA staining of nuclei. Confocal microscopy was performed with a Zeiss LSM-510 (Carl Zeiss Micro-Imaging, Jena, Germany) system. Alexa-fluor-555 was excited with a helium/neon laser (He/Ne) at 543 nm emitting fluorescence at maximum 565 nm. DAPI was excited with a diode laser at 405 nm emitting fluorescence at maximum 454 nm.

**In silico analysis**

The ten Tusscher model simulates stimulus-triggered, whole-cell APs for endocardial, epicardial and M-cell myocytes. For endocardial cells, maximal Ito conductance is reduced to 75% and Ito kinetics are voltage-shifted by about -20 mV, producing AP morphology with a less prominent notch at the end of phase 1 relative to epicardial and M-cells. For M-cells, maximal I_Ks_ conductance is reduced to 75% relative to epicardial and endocardial cells, resulting in M-cell AP duration (APD) being much longer (>100 ms longer at 1 Hz) than that of epicardial or endocardial cells. This model closely replicates experimentally observed APD restitution curves and demonstrates APD alternans with an increasing slope of AP restitution at rapid pacing cycle lengths (<250 ms).[6,7]

**Table 1**

**Primers for PCR**

| **Primers** | **Forward** | **Reverse** |
| --- | --- | --- |
| mutation-containing primer | AGATCCATCAGGACGACCTGCT | GTCGTCCTGATGGATCTTGTGTAG |
| flanking primer | CACCAACGGCATCGACATGAACG | GTGTCGCTGCTCTTCTCGCAGTC |

**References**

1. Flicek P, Aken BL, Beal K, Ballester B, Caccamo M, Chen Y, Clarke L, Coates G, Cunningham F, Cutts T, Down T, Dyer SC, Eyre T, Fitzgerald S, Fernandez-Banet J, Graf S, Haider S, Hammond M, Holland R, Howe KL, Howe K, Johnson N, Jenkinson A, Kahari A, Keefe D, Kokocinski F, Kulesha E, Lawson D, Longden I, Megy K, Meidl P, Overduin B, Parker A, Pritchard B, Prlic A, Rice S, Rios D, Schuster M, Sealy I, Slater G, Smedley D, Spudich G, Trevanion S, Vilella AJ, Vogel J, White S, Wood M, Birney E, Cox T, Curwen V, Durbin R, Fernandez-Suarez XM, Herrero J, Hubbard TJ, Kasprzyk A, Proctor G, Smith J, Ureta-Vidal A, Searle S (2008) Ensembl 2008. Nucleic Acids Res 36: D707-D714.

2. Adzhubei IA, Schmidt S, Peshkin L, Ramensky VE, Gerasimova A, Bork P, Kondrashov AS, Sunyaev SR (2010) A method and server for predicting damaging missense mutations. Nat Methods 7: 248-249.

3. Kumar P, Henikoff S, Ng PC (2009) Predicting the effects of coding non-synonymous variants on protein function using the SIFT algorithm. Nat Protoc 4: 1073-1081.

4. Ehrlich JR, Pourrier M, Weerapura M, Ethier N, Marmabachi AM, Hebert TE, Nattel S (2004) KvLQT1 modulates the distribution and biophysical properties of HERG. A novel alpha-subunit interaction between delayed rectifier currents. J Biol Chem 279: 1233-1241.

5. Biliczki P, Girmatsion Z, Harenkamp S, Anneken L, Brandes RP, Varro A, Marschall C, Herrera D, Hohnloser SH, Nattel S, Ehrlich JR (2008) Cellular properties of C-terminal KCNH2 long QT syndrome mutations: description and divergence from clinical phenotypes. Heart Rhythm 5: 1159-1167.

6. ten Tusscher KH, Noble D, Noble PJ, Panfilov AV (2004) A model for human ventricular tissue. Am J Physiol Heart Circ Physiol 286: H1573-H1589.

7. ten Tusscher KH, Panfilov AV (2006) Alternans and spiral breakup in a human ventricular tissue model. Am J Physiol Heart Circ Physiol 291: H1088-H1100.
